# Superficial Dorsal Vein Thrombosis of the Penis and Pulmonary Embolism in a 15-year-old Boy: A Case Report

**DOI:** 10.5811/cpcem.21138

**Published:** 2024-12-06

**Authors:** Tomas Leng, Jason H. Homme, Jana Anderson

**Affiliations:** * Mayo Clinic, Department of Pediatric and Adolescent Medicine, Rochester, Minnesota; † Mayo Clinic, Department of Emergency Medicine, Rochester, Minnesota

**Keywords:** penile pain, thrombosis, pediatrics, pulmonary embolism

## Abstract

**Introduction:**

Penile pain in children and adolescents is an uncommon presenting symptom in the emergency department (ED). The differential diagnosis includes trauma, priapism, urethral stone, infection, Mondor disease, Peyronie disease, and thrombosis.

**Case Report:**

A 15-year-old male with a high-risk, B-cell acute lymphocytic leukemia and recent pegaspargase administration presented to the ED with new-onset penile pain. After the administration of opioid analgesics, he developed hypoxia prompting an urgent computed tomography pulmonary angiogram that revealed bilateral segmental acute pulmonary embolism (PE). Ultrasound of the penis revealed findings consistent with superficial dorsal vein thrombosis of the penis.

**Conclusion:**

To our knowledge, this is the first case report of an adolescent with superficial dorsal vein thrombosis of the penis and a coexisting PE. Doppler ultrasound can provide a prompt assessment of penile induration and differentiate venous thrombosis from other causes.

## INTRODUCTION

Penile pain in children and adolescents is an uncommon presenting symptom in the emergency department (ED). The differential diagnosis of acute penile pain in the ED should include trauma, priapism, urethral stone, infection, Mondor disease, and Peyronie disease.^1^ A growing body of evidence suggests that Doppler ultrasound (US) is the imaging method of choice for the investigation of vascular causes of penile pain.^2^ For patients with acute severe pain, parenteral opioids are commonly first-line therapy.^3^ Although a decrease in oxygen saturation is a well-described effect of acute opioid administration, it is usually transient and should not distract from the evaluation for pulmonary embolism (PE) in a patient with prothrombotic risk factors.^4^ We present a case of an adolescent male with penile pain with superficial dorsal vein thrombosis of the penis who was found to have a coexisting PE.

## CASE REPORT

A 15-year-old male with a medical history significant for high-risk B-cell acute lymphoblastic leukemia (ALL) and on maintenance chemotherapy presented to clinic for his scheduled chemotherapy, which was deferred due to his report of new-onset, intense penile pain. A limited physical exam revealed an indurated shaft of the penis that was tender to palpation. A complete blood cell count revealed a hemoglobin of 8.7 grams/deciliter (g/dL) (reference range for age: 13.3–16.9 g/dL) and a platelet count of 73 × 10^9^/liter (L) (139–320 × 10^9^/L). The white cell count was 2.9 × 10^9^/L (3.8–10.4 × 10^9^/L) with absolute neutrophil count of 0.58 × 10^9^/L (1.40–6.10 × 10^9^/L). A swab performed for SARS-CoV-2 was negative. Urinalysis was within normal limits, and a urine culture was sent. Due to his excruciating penile pain, the patient was referred to the ED for further evaluation.

Upon arrival to the ED, the patient was in acute distress due to the severity of his pain, rated as 9/10. He was alert and speaking in complete sentences without respiratory distress. He confirmed acute onset of penile pain lasting for the prior 18 hours with progressive worsening. Initial vital signs were recorded as temperature 36.9° Celsius, heart rate 89 beats per minute (BPM), blood pressure 123/75 millimeters of mercury, respiratory rate 22 breaths per minute, and oxygen saturation 95% on room air. He received fentanyl (1 microgram per kilogram [kg]) soon after arrival, which decreased his pain level to 2/10. The patient denied any recent trauma, risk factors or concern for sexually transmitted infections or abuse. He reported decreased urinary output and urinary leakage, with darker appearing urine. The pain was described as constant and exacerbated by urination or movement of the penile shaft. He reported decreased physical activity and mobility in the setting of his high-risk B-cell ALL and chemotherapy. He had a history of COVID-19 infection seven months prior but no other significant recent infections.

On genital examination, his circumcised penis was noted to have mild erythema and marked swelling and induration of the shaft most prominent in the dorsal region of the penis as well as tenderness to palpation. The glans of the penis appeared normal. Testicular and scrotal examination were normal. He had an occasional wet cough, but his lungs were clear to auscultation. There was no splenomegaly, and all other routine exam findings were normal. Due to worsening penile pain, the patient received phenazopyridine and morphine 4 milligrams (mg). Pediatric urology was consulted and recommended an evaluation for urolithiasis with a kidney, ureter, and bladder radiograph and kidney US, which was negative.

**Image 1:**
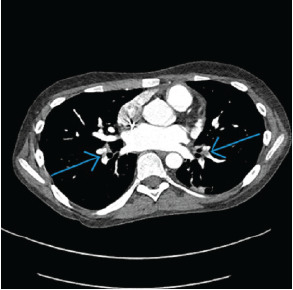
Computed tomography pulmonary angiogram demonstrating pulmonary emboli in the right middle lobe and left upper lobe (blue arrows).

**Image 2:**
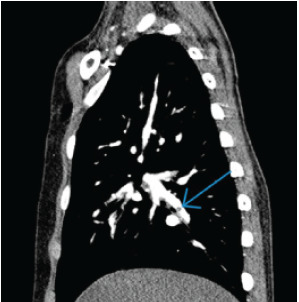
Computed tomography pulmonary angiogram demonstrating a segmental pulmonary embolus (blue arrow).

Approximately 2½ hours after morphine administration, the patient reported improved pain; however, his oxygen saturation decreased intermittently to 75% and improved to 95% with 1 L per minute of oxygen via nasal cannula. Upon reexamination by a second clinician, the patient’s heart rate was noted to have increased to 100 BPM at rest despite improved pain control. A chest radiograph demonstrated mild perihilar interstitial opacities and central pulmonary vascular congestion. Due to continued intermittent desaturations to 75%, tachycardia, and prothrombotic risk factors of ALL and immobility, a computed tomography pulmonary angiogram (CTPA) was obtained. It showed bilateral segmental acute PE involving multiple segmental right and left pulmonary arteries (Images 1, 2) and left lower lobe atelectasis.

**CPC-EM Capsule**

What do we already know about this clinical entity?

*Penile Mondor disease (PMD) is a self-limiting thrombophlebitis of the superficial dorsal penile vein that should be considered in the differential diagnosis of penile pain*.

What makes this presentation of disease reportable?

*To our knowledge, this is the first reported case of PMD and co-occurring pulmonary embolism*.

What is the major learning point?

*Doppler ultrasound can provide a prompt assessment of penile induration and differentiate PMD from other causes*.

How might this improve emergency medicine practice?

*Early identification of PMD may allow for the rapid, noninvasive diagnosis of penile thrombosis and guide disposition*.

Point-of-care ultrasound of the penis performed by an experienced emergency physician revealed a non-compressible dorsal vein and internal heterogenous echogenicity in the superficial dorsal vein of the penis suggestive of a thrombus. Color Doppler US revealed reduced venous flow signals in this region. Bilateral dorsal arterial flow signals were normal. An electrocardiogram was normal. The CBC revealed thrombocytopenia with a platelet count of 54 × 10^9^/L. Prothrombin time was 17.5 seconds (9.4–12.5 seconds) with international normalized ratio of 1.6 (0.9–1.1). N-terminal pro B-type natriuretic peptide was 120 picograms/milliliter (pg/mL) (≤158 pg/mL); and troponin T was 9 nanograms (ng)/L (≤15 ng/L).

The patient was admitted to the pediatric hematology-oncology service and started on unfractionated heparin. An echocardiogram was performed and did not show any evidence of heart strain. Ultrasound of the upper and lower extremities was also performed without evidence of thrombi. The patient’s pain improved after initiation of heparin infusion and completely resolved after 48 hours. Repeat US of the penis demonstrated resolution of the thrombosis consistent with the observed clinical improvement. After three days of a heparin infusion, the patient was discharged home on subcutaneous enoxaparin 1 mg/kg/day divided twice a day with close follow-up. He reported no recurrence of additional concerns at a follow-up visit three days after discharge.

## DISCUSSION

This case of superficial dorsal vein thrombosis of the penis and concomitant PE highlights the importance of considering vascular etiologies such as thrombosis in patients with prothrombotic risk factors who present with penile pain. Penile venous thrombosis typically presents with episodic or continuous throbbing pain, and erythema and edema may also be present as was the case for our patient.^5^ Generally, a superficial dorsal vein thrombosis of the penis has been described in the literature as penile Mondor disease (PMD).^6^ It is a rare and self-limiting superficial dorsal vein thrombophlebitis that has been described in young and middle-aged, sexually active men as well as in recent SARS-CoV-2 positive patients with cardiovascular disease.^7^ It typically manifests as a visible painful cord located along the dorsal surface of the penis. This contrasts with the findings in our patient who had more generalized shaft induration and urinary dribbling, which would be atypical for a case of PMD. Unlike our patient who presented with acute pain, most cases of PMD described in the literature typically presented with progressive pain on the dorsum of the penis lasting for a few days, which was exacerbated by erection.^8^

While penile venous thrombosis can be suspected based on a medical history and physical examination, color Doppler US plays an important role in differentiating it from other conditions. Non-compressibility of the dorsal vein with no flow inside the vein on color or spectral Doppler studies should raise concern for thrombosis. Although US of the lower extremities is the first-line modality to evaluate for thrombosis in major veins, it often cannot effectively visualize venous drainage above the inguinal ligament, which might have been an originating site of the penile thrombus in our case. In such cases, considering a computed tomography of the abdomen and pelvis (CTPA) may reveal the site of deep venous thrombosis that can potentially be responsible for venous thromboembolism at other sites. Segmental PEs alone identified on the CTPA would probably not account for the hypoxia seen in our patient. However, the finding of the left lower lobe atelectasis on the CTPA, along with a suspected viral infection and the administration of opioids, likely contributed to the patient’s hypoxia. Selection of additional appropriate imaging modalities should be guided by the severity and complexity of a patient’s clinical condition.

Management in the ED should focus on pain control and identifying an underlying cause. In most instances of Mondor disease, supportive care and expectant management are usually adequate. Initial treatment typically includes applying warm compresses, nonsteroidal anti-inflammatory drugs, and avoiding irritating clothing or activities. Although the use of anticoagulation for PMD is controversial, some studies have reported the effectiveness of anticoagulation in the acute phase using prophylactic or intermediate doses of low-molecular-weight heparin and subcutaneous administration of fondaparinux.^9^ The main factors prompting heparin administration and admission in our patient were his risk factors and the co-existing PE. Ultimately, the decision to anticoagulate and admit the patient should be made on a case-by-case basis. Most patients with isolated PMD can be discharged from the ED safely and should be advised to abstain from sexual activity until thrombus resolution. In most previously reported cases, the resolution of thrombosis varied from two to eight weeks.^10^ Topical heparin-containing creams, thrombectomy, and superficial penile vein resection have been reported as treatment modalities for subacute and chronic cases of PMD.

Venous thromboses in unusual sites in hypercoagulable patients are rare and may involve any portion of the venous system. It should be noted that the PEs in our patient were not from the dorsal vein of the penis but rather from the hypercoagulable state. The prothrombotic state of malignancy and the addition of pegaspargase likely predisposed our patient to thromboemboli. Pegaspargase is known to decrease the levels of anticoagulant proteins C, S and antithrombin III, increasing the risk of thrombosis.^4^ The differential diagnosis of acute penile pain in the ED includes trauma, priapism, urethral stone, infection, and acute worsening of chronic conditions such as sclerosing lymphangitis and Peyronie disease. In all cases, a detailed physical examination and history should provide diagnostic cues.

## CONCLUSION

Superficial dorsal vein thrombosis of the penis is a rare cause of acute penile pain in the ED that poses a diagnostic challenge. Vascular etiologies of penile pain in the ED should always be considered, and risk factors may point toward underlying etiology. Doppler ultrasound is the first-line imaging modality in evaluation of penile induration and can differentiate venous thrombosis from other etiologies.
